# Transkorneale Elektrostimulation bei Retinitis pigmentosa: Prüfplan einer multizentrischen, prospektiven, randomisierten, kontrollierten und doppelblinden Studie im Auftrag des Gemeinsamen Bundesausschusses (G-BA-Erprobungsrichtlinie)

**DOI:** 10.1007/s00347-021-01360-1

**Published:** 2021-03-19

**Authors:** Nadine Kahle, Tobias Peters, Angelika Braun, Jeremy Franklin, Claudia Michalik, Florian Gekeler, Barbara Wilhelm, retina.net e. V., TES-RP-Studiengruppe, Antonios Koutsonas, Antonia Joussen, Klaus Rüther, Frank G. Holz, Thomas Kohnen, Fanni Molnar, Martin Spitzer, Klaus Rohrschneider, Johann Roider, Tim Krohne, Catarina Busch, Katrin Lorenz, Claudia Priglinger, Ulrich Kellner, Florian Gekeler, Peter Szurmann, Katarina Stingl, Jost Hillenkamp

**Affiliations:** 1 Department für Augenheilkunde, Universitätsklinikum Tübingen, Elfriede-Aulhorn-Straße 7, 72076 Tübingen, Deutschland; 2 Institut für Medizinische Statistik und Bioinformatik, Medizinische Fakultät, Universität zu Köln, Köln, Deutschland; 3 Zentrum für Klinische Studien, Medizinische Fakultät, Universität zu Köln, Köln, Deutschland; 4 Klinikum der Landeshauptstadt Stuttgart gKAöR, Stuttgart, Deutschland; 5 Steinbeis Transfer Zentrum (STZ) eyetrial am Department für Augenheilkunde, Universitätsklinikum Tübingen, Tübingen, Deutschland

**Keywords:** Retinadystrophien, Retinadegeneration, Netzhauterkrankungen, Elektrische Stimulationstherapie, Gesichtsfeld, Retinal dystrophies, Retinal degeneration, Retinal diseases, Electric stimulation therapy, Visual field

## Abstract

Der Schwerpunkt dieser großen, multizentrischen Erprobungsstudie im Auftrag des Gemeinsamen Bundesausschusses (G-BA) liegt in der Feststellung eines Nutzens der transkornealen Elektrostimulation für Patienten mit Retinitis pigmentosa (RP). Hauptkriterium ist das kinetische Gesichtsfeld und die Frage, ob die Verschlechterung der Studienaugen im Vergleich zu den zum Schein (sham‑)stimulierten Partneraugen über einen Behandlungszeitraum von 3 Jahren langsamer fortschreitet.

## Abstract (en)

The focus of this large multicenter trial commissioned by the Joint Federal Committee (*Gemeinsamer Bundesausschuss*, G‑BA) is to determine a benefit of transcorneal electrical stimulation for retinitis pigmentosa (RP) patients. The main criterion for benefit is the kinetic visual field and whether the deterioration progresses more slowly in the study eyes compared to the sham-stimulated fellow eyes over a treatment period of 3 years.

## Erprobungsregelung des Gemeinsamen Bundesausschusses

Bei der Bewertung, ob medizinische Untersuchungs- und Behandlungsmethoden durch die Krankenkassen erstattet werden, hat der Gemeinsame Bundesausschuss (G-BA) jeweils den aktuellen Stand der medizinischen Erkenntnisse und wissenschaftlichen Datenlage zu berücksichtigen.

Im Fall der transkornealen Elektrostimulation (TES) ist die Studienlage aktuell für eine abschließende Beurteilung des Nutzens dieser Methode noch nicht ausreichend. Die bisherigen Ergebnisse präklinischer und klinischer Studien weisen darauf hin, dass die TES das Potenzial einer nutzenbringenden Behandlungsmethode hat [5–8]. Daher soll diese Methode im Rahmen einer Erprobungsregelung als Studie getestet werden. Mit der Gesamtkoordination des Projekts wurde das Universitätsklinikum Tübingen (UKT) beauftragt, als Partner des UKT sind retina.net e. V., das Steinbeis Transfer Zentrum (STZ) *eyetrial* am Department für Augenheilkunde des Universitätsklinikums Tübingen und das Zentrum für klinische Studien (ZKS) der Universität zu Köln beteiligt.

Im Folgenden werden die Hintergründe sowie der für die Erprobungsregelung entwickelte Prüfplan zur geplanten Studie, die im Frühjahr 2021 beginnen wird, vorgestellt.

## Transkorneale Elektrostimulation

### Durchführung

Das OkuStim®-System (Okuvision GmbH, Reutlingen), mit dem die transkorneale Elektrostimulation (TES) durchgeführt wird, ist ein zugelassenes und CE-gekennzeichnetes Medizinprodukt, das seit mehreren Jahren von Patienten mit Retinitis pigmentosa (RP) angewendet wird. Bisher erfolgte die Therapie auf Kosten des Patienten oder in Einzelfall-Entscheidungen finanziert durch die Krankenkassen.

**Figure 1:**
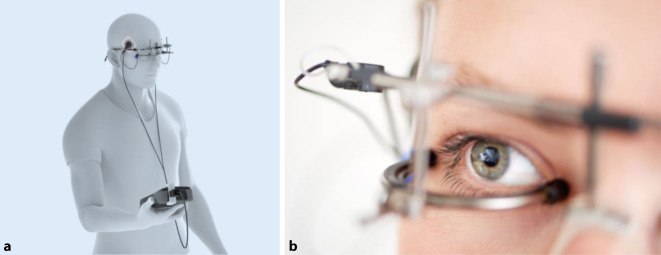

OkuStim® besteht aus 3 Komponenten: OkuStim®-Handgerät, OkuSpex-Brille® und OkuEl®-Elektroden (Abb. 1). Die OkuSpex®-Brille dient als Halterung für die OkuEl®-Elektroden und kann individuell an die Gesichtsform des Patienten angepasst werden. Die OkuEl®-Elektroden sind so gestaltet, dass sie nur die Bindehaut berühren und so die elektrischen Impulse auf die Augen übertragen können. Im Schläfenbereich wird die Gegenelektrode angebracht. Über die Brille werden die Elektroden stets an beiden Augen angelegt. Den Patienten wird nicht mitgeteilt, welches Auge stimuliert wird und als Studienauge gilt. Bei der Sham-Prozedur wird nach einem initial beidseitigen Stimulationsreiz eine Seite (Auge) „abgeschaltet“ und somit nicht mehr stimuliert. Der Patient hat so den Eindruck einer Stimulation beider Augen bzw. kann nicht zuordnen, welches Auge behandelt wird (Verblindung).

### Wirkung

Vorangegangene präklinische und klinische Studien zeigten positive Trends und teils signifikante Ergebnisse, die darauf hinweisen, dass die TES ein vielversprechender Therapieansatz für Patienten mit RP sein kann [6–8]. Die Wirkungsweise der Elektrostimulation wurde dabei bisher auf folgende Mechanismen, oder eine Kombination aus mehreren dieser Mechanismen, zurückgeführt [10]: Regulation neurotropher [6], vasodilatatorischer [1, 2, 4] sowie antiapoptotischer und antientzündlicher Faktoren und Mechanismen [11]. Um diese Effekte aufrechtzuerhalten, scheint eine dauerhafte Therapie notwendig zu sein [9].

Während der Durchführung der bisher veröffentlichten klinischen Studien traten keine schweren Nebenwirkungen auf. Häufige, vorübergehende Beschwerden sind einfach zu behandelnde trockene Augen.

Insgesamt kommen die bisherigen Studien zu dem Schluss, dass größere Patientenzahlen und längere Beobachtungszeiten erforderlich sind, um ergänzende, belastbare Daten zu erhalten und die positiven Effekte der Elektrostimulation, also die Verlangsamung des Krankheitsverlaufs, gemessen am Gesichtsfeld, bei RP-Patienten zeigen zu können. Diese Evidenzlücke soll mit der geplanten Studie geschlossen werden.

### Indikation

**Figure 2:**
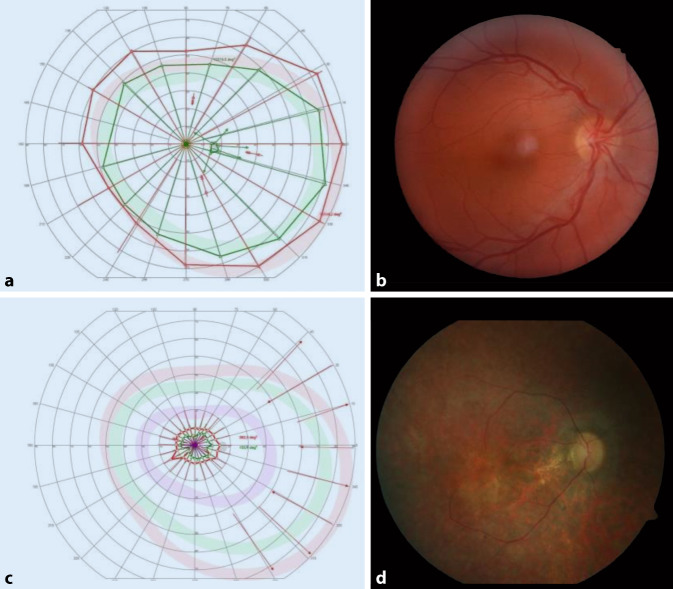

Retinitis pigmentosa (RP) bezeichnet eine Familie erblicher Netzhautdystrophien mit einer durchschnittlichen Prävalenz von 2,2/10.000 in Europa. Sie ist gekennzeichnet durch eine fortschreitende Netzhautdegeneration, die in der Peripherie beginnt und sich allmählich zum Zentrum der Netzhaut hin ausdehnt, wodurch sich das Gesichtsfeld im Krankheitsverlauf stetig verringert. In Abb. 2 sind die Unterschiede zwischen Gesichtsfeldmessung und Fundusfoto eines gesunden Probanden (Abb. 2a, b) im Vergleich zu jenen eines RP-Patienten (Abb. 2c, d) dargestellt.

Beginnend oft mit Nachtblindheit im ersten oder zweiten Lebensjahrzehnt, kommt es meist im dritten und vierten Lebensjahrzehnt zum Verlust des peripheren Sehens (Tunnelblick) bis hin zur vollständigen Erblindung im Spätstadium. Die RP stellt eine der häufigsten Ursachen für Blindheit oder schwere Sehschwäche bei Menschen im Alter von 20 bis 60 Jahren dar [3].

Dass die Patienten aufgrund des fortschreitenden Verlaufs der Krankheit Schritt für Schritt ihr Sehvermögen verlieren und es derzeit noch – mit Ausnahme der Gentherapie einer einzigen Mutation – keine zugelassene Therapie gibt, die den Krankheitsverlauf verlangsamen oder stoppen kann, ist sowohl für die Betroffenen als auch für die behandelnden Ärzte eine große Herausforderung und Belastung. Deshalb ist es von großer Relevanz, effektive Behandlungsmethoden für diese zur Blindheit führende Krankheit zu entwickeln.

## Kriterien für die Studienteilnahme

### Einschlusskriterien

1. Patienten mit syndromaler oder nichtsyndromaler RP (autosomal-dominant, autosomal-rezessiv und „x-linked“),
2. Erwachsene jeden Geschlechts im Alter von 18–80 Jahren,
3. bestkorrigierte Sehschärfe (ETDRS-Visus, gemäß Early Treatment Diabetic Retinopathy Study) zwischen 0,1 und 0,8 in beiden Augen mit nicht mehr als 15 ETDRS-Buchstaben (3 Zeilen) Unterschied zwischen den Augen,
4. Octopus 900 (Fa. Haag-Streit, Wedel), kinetisches Gesichtsfeld:
  - Gesichtsfeldfläche ≥75 deg^2^ (entspricht einem durchschnittlichen Radius von 5 Grad; Stimulus V4e) in beiden Augen,
  - der Unterschied zwischen beiden Augen darf eine Ratio „OD/OS“ (Oculus dexter/Oculus sinister) von nicht unter 0,5 und nicht mehr als 2 aufweisen,
5. Patienten in der Lage und willens, das schriftliche Einverständnis zur Studie zu geben.

### Wichtigste ophthalmologische Ausschlusskriterien

1. Optikusneuritis/Optikusneuropathie,
2. diabetische Retinopathie,
3. Neovaskularisationen der Netzhaut unterschiedlicher Genese,
4. Zustand nach retinalem Arterien- oder Venenverschluss,
5. Zustand nach Netzhautablösung oder nach vitreoretinaler Chirurgie,
6. Silikonöltamponade,
7. trockene oder neovaskuläre altersbedingte Makuladegeneration (AMD),
8. Makulaödem,
9. Glaukom,
10. Hornhautdegeneration mit Auswirkung auf die Sehschärfe,
11. Trübung der brechenden Medien, die eine optische Kohärenztomographie (OCT)/Ophthalmoskopie beeinträchtigen,
12. Katarakt, die nach Einschätzung des Prüfers im Studienzeitraum operationsbedürftig werden kann,
13. Aphakie,
14. TES-Therapie in den letzten 3 Monaten (Teilnahme ist nach einer 3‑monatigen Therapiepause möglich).

## Studienablauf und Untersuchungen

Ab Frühjahr 2021 werden deutschlandweit 154 Patienten an 18 deutschen Studienzentren rekrutiert. Pro Patient beträgt die Studiendauer 3 Jahre und umfasst insgesamt 8 Termine, zu denen die Patienten an die Prüfzentren kommen. In einer Screening-Visite werden zunächst alle Ein- und Ausschlusskriterien überprüft.

Jeder Patient und seine Angehörigen erhalten zu Beginn der Studie an der Klinik eine genaue Einweisung in die Anwendung und Handhabung des OkuStim®-Systems, um dieses während der Studie selbstständig und studienkonform zu Hause selbst anwenden zu können.

Die Stimulationsdauer beträgt 30 min einmal wöchentlich über einen Zeitraum von 3 Jahren. Die Stimulationsstärke ist auf 0,8 mA festgelegt und wird durch das Handgerät mit biphasischen Pulsen und einer Pulsfrequenz von 20 Hz bereitgestellt. Sollten bei der Behandlung mit 0,8 mA Missempfindungen auftreten, kann die Stimulationsstärke auf 0,6 mA reduziert werden.

Im Rahmen der Studie wird nur ein zufällig ausgewähltes Auge stimuliert, das Partnerauge dient als Kontrolle und erhält eine Scheinbehandlung. Weder dem Patienten noch dem Studienpersonal inklusive der Ärzte ist bekannt, welches Auge stimuliert wird (randomisiertes, doppelblindes Studiendesign).

Um eine Verminderung des relativen Gesichtsfeldverlusts im stimulierten Auge, verglichen mit dem relativen Gesichtsfeldverlust im zum Schein (sham‑)behandelten Auge, bewerten zu können, werden kinetische Gesichtsfelduntersuchungen mit dem Octopus 900 Pro (Fa. Haag-Streit, Wedel) zu 4 Zeitpunkten durchgeführt. Dabei dienen die beiden bei der Screening-Visite durchgeführten Gesichtsfelduntersuchungen der Minimierung von Lerneffekten. Sie finden in der statistischen Analyse des primären Endpunkts keine Verwendung. Weitere Untersuchungsmethoden sind neben der augenärztlichen Routineuntersuchung die spektrale optische Kohärenztomographie („spectral-domain optical coherence tomography“, SD-OCT), die Kontrastempfindlichkeit (Pelli-Robson-Tafel), die dunkeladaptierte Empfindlichkeitsschwelle („full-field stimulus testing“, FST) und der Roth-28-Hue-Test sowie die Befragung anhand des Visual Function Questionnaire 25 (VFQ-25). Alle Studiendaten werden in einer elektronischen Datenbank (elektronischer Prüfbogen, „electronical case report form“, eCRF) gesammelt und am Studienende statistisch ausgewertet.

Das Sicherheitskomitee (Data Safety Monitoring Board; DSMB) setzt sich aus 2 erfahrenen Ophthalmologen und einem Statistiker zusammen und berät regelmäßig anhand von Sicherheitskriterien über die Fortsetzung der Studie.

## Ziel und Endpunkte der Studie

Ziel dieser Studie ist es, den Nutzen der TES als Behandlungsmethode bei Retinitis pigmentosa (RP) zu prüfen.

### Primärer Endpunkt

Verminderung des Flächenverlusts im kinetischen Gesichtsfeld (Prüfmarke V4e; gemessen als Differenz zwischen Baseline und 3‑Jahres-Visite) des behandelten im Verhältnis zum sham-behandelten Auge. Dabei gilt als Ausgangs- bzw. Endwert der Mittelwert von je 2 Gesichtsfeld(GF)-Untersuchungen bei der Baseline-Visite bzw. nach 3 Jahren Behandlung.

### Sekundäre Endpunkte

- Kontrastempfindlichkeit,
- bestkorrigierte Sehschärfe (BCVA, „best-corrected visual acuity“),
- statisches Gesichtsfeld,
- Farbsinn,
- Netzhautmorphologie,
- Anzahl und Schweregrad unerwünschter Ereignisse,
- sehbezogene Lebensqualität.

## Fazit für die Praxis

- In einer Multizenterstudie wird die Wirksamkeit der transkornealen Elektrostimulation bei Retinitis pigmentosa (RP) geprüft.
- Über 150 RP-Patienten werden an 18 deutschen Augenkliniken/Praxen 3 Jahre lang unter Therapie mit transkornealer Elektrostimulation (TES; einmal wöchentlich 30 min) beobachtet.
- Vor allem das kinetische Gesichtsfeld soll zeigen, ob im Vergleich zum Kontrollauge das behandelte Auge einen deutlich geringeren Gesichtsfeldverlust aufweist.
- Nach Studienauswertung wird der Gemeinsame Bundesausschuss (G-BA) den Nutzen der TES-Therapie abschließend bewerten.
